# Music genetics research: Association with musicality of a polymorphism in the AVPR1A gene

**DOI:** 10.1590/1678-4685-GMB-2016-0021

**Published:** 2017-05-22

**Authors:** Luiza Monteavaro Mariath, Alexandre Mauat da Silva, Thayne Woycinck Kowalski, Gustavo Schulz Gattino, Gustavo Andrade de Araujo, Felipe Grahl Figueiredo, Alice Tagliani-Ribeiro, Tatiana Roman, Fernanda Sales Luiz Vianna, Lavínia Schuler-Faccini, Jaqueline Bohrer Schuch

**Affiliations:** 1 Programa de Pós-Graduação em Genética e Biologia Molecular, Departamento de Genética, Universidade Federal do Rio Grande do Sul, Porto Alegre, RS, Brazil; 2 Programa de Pos-Graduação em Saúde da Criança e Adolescente, Faculdade de Medicina, Universidade Federal do Rio Grande do Sul, Porto Alegre, RS, Brazil

**Keywords:** musicality, music, vasopressin, AVPR1A

## Abstract

Musicality is defined as a natural tendency, sensibility, knowledge, or talent to create, perceive, and play music. Musical abilities involve a great range of social and cognitive behaviors, which are influenced by both environmental and genetic factors. Although a number of studies have yielded insights into music genetics research, genes and biological pathways related to these traits are not fully understood. Our hypothesis in the current study is that genes associated with different behaviors could also influence the musical phenotype. Our aim was to investigate whether polymorphisms in six genes (AVPR1A, SLC6A4, ITGB3, COMT, DRD2 and DRD4) related to social and cognitive traits are associated with musicality in a sample of children. Musicality was assessed through an individualized music therapy assessment profile (IMTAP) which has been validated in Brazil to measure musical ability. We show here that the RS1 microsatellite of the AVPR1A gene is nominally associated with musicality, corroborating previous results linking AVPR1A with musical activity. This study is one of the first to investigate musicality in a comprehensive way, and it contributes to better understand the genetic basis underlying musical ability.

## Introduction

The complex behavior of humans is closely influenced by genetic and environmental factors. An increasing number of studies have focused on the biological basis of these characteristics and, currently, many biological pathways and genes involved in different human behaviors are known (Ebstein *et al.*, 2009). Among several factors previously studied, some neuropeptides (*e.g.*, vasopressin) seem to play an important role in affiliative behaviors and social recognition. Serotonergic and dopaminergic neurotransmitter systems are also associated with social cognition and emotional behavior (*e.g.*, anxiety and mood) (Skuse and Gallagher, 2011).

Musicality is described as a natural tendency, sensibility, or talent to create, perceive, and play music. A great range of behavioral, social, and cognitive traits are involved in musical abilities such as playing instruments, singing, dancing, arranging music, and improvising (Ukkola *et al.*, 2009). Thus, it is possible that the same factors involved in behavioral traits could also influence musical aptitudes. A number of studies have begun to unveil the genetic basis underpinning musicality, with some promising findings being replicated (see the review by Tan *et al.*, 2014). Despite these recent insights, the genetic basis involved in this trait is not fully understood.

Recently, genomic approaches have been applied to identify genetic loci associated with music skills (see the review by Gingras *et al.*, 2015). Genome-wide linkage and association analyses have pointed several genetic variants which could be segregating with musical aptitude. In particular, a region on chromosome 4q22-23 was identified in two independent linkage-mapping studies in Finnish and Mongolian populations (Pulli *et al.*, 2008; Park *et al.*, 2012; Oikkonen and Jarvela, 2014). Most recently, genome-wide linkage and association scan identified multiple loci containing genes affecting auditory and cognitive functions (Park *et al.*, 2012; Gingras *et al.*, 2015; Oikkonen *et al.*, 2015). These studies suggested that variants in *UGT8* (UDP glycosyltransferase 8), *GSG1L* (Germ Cell-Specific Gene 1-Like) and *UNC5C* (unc-5 netrin receptor C) genes, might be involved in musical phenotypes (Park *et al.*, 2012; Gingras *et al.*, 2015; Oikkonen *et al.*, 2015). Additionally, copy number variations have been associated with musical abilities, revealing interesting regions involved in neurodevelopment, learning and memory (Ukkola-Vuoti *et al.*, 2013).

In candidate gene association studies, the major findings are related to vasopressin, serotonin and dopamine genes, due to their roles on different behavioral and cognitive traits (Reuter *et al.*, 2006; Donaldson and Young, 2008). Concerning these studies, arginine vasopressin receptor 1A (*AVPR1A*) and serotonin transporter (*SLC6A4*) are the main genes associated with musical activities and related behaviors (Bachner-Melman *et al.*, 2005; Granot *et al.*, 2007; Ukkola *et al.*, 2009; Ukkola-Vuoti *et al.*, 2011). Other genes, such as *TPH1, COMT, DRD2, DRD5, IGF2, MAOA* (Bachner-Melman *et al.*, 2005; Ukkola *et al.*, 2009; Tan *et al.*, 2014) have also been investigated in some of these studies, but the findings were inconclusive.

*AVPR1A* (12q14-q15) encodes a neuropeptide that is responsible for mediating the influence of the hormone arginine vasopressin (AVP) in the brain. It has an important role in the control of cognitive functions, influencing memory and learning, as well as acting in the modulation of social behavior (Fink *et al.*, 2007). The highly variable microsatellites RS1, RS3, and AVR have been studied extensively in different behavioral traits such as social cognitive behavior (Israel *et al.*, 2008), and autism spectrum disorders (Wassink *et al.*, 2004). Among musical characteristics, these *AVPR1A* microsatellites have been associated with music memory (Granot *et al.*, 2007), musical perception (Ukkola *et al.*, 2009) and music listening (Ukkola-Vuoti *et al.*, 2011).

The serotonergic system has been indicated as an important modulator in the control of several behavioral and physiological functions (Coiro *et al.*, 2010). *SLC6A4* (17q11.2) codes for the serotonin transporter and is found in the brain, mostly in the cortex and limbic systems, which are both involved with emotions (Ukkola *et al.*, 2009). The 5-HTTLPR, which is a functional polymorphism located in the promoter region of the gene, influences the rate of serotonin transcription and is associated with different behaviors, including emotional (Zalsman *et al.*, 2006; Cools *et al.*, 2008; Petersen *et al.*, 2012) and musical characteristics such as music memory (Granot *et al.*, 2007) and creative dance performance (Bachner-Melman *et al.*, 2005). Another gene involved with this system is integrin β3 (*ITGB3*). *ITGB3* (17q21.32), which encodes a protein involved in neural cellular adhesion and has been characterized as a quantitative trait locus for serotonin levels in blood (Weiss *et al.*, 2004). Some studies have demonstrated that variants in this gene are associated with an increase in serotonin transporter expression and in serotonin uptake (Carneiro *et al.*, 2008; Whyte *et al.*, 2014). These variants have also been related to behavioral disorders (Weiss *et al.*, 2006a; Napolioni *et al.*, 2011; Schuch *et al.*, 2014).

Brain dopamine neurotransmitters are important in the modulation of sensory and motor responses as well as for executive and cognitive functions, and they have been associated with different behavioral traits (Cools and D’Esposito, 2011). Catechol-O-methyltransferase (COMT) is an enzyme that is essential for degrading dopamine in the prefrontal cortical area. It is involved in the regulation of brain dopamine levels. The functional polymorphism Val158Met in the *COMT* gene (22q11.21) influences the modulation of COMT activity (Wang *et al.*, 2013), and it has been associated with memory, intelligence, and emotional difficulties (Tunbridge *et al.*, 2006; Shaw, 2007).

Five different receptor subtypes mediate dopamine action; however, dopamine receptor D2 (*DRD2*) and dopamine receptor D4 (*DRD4*) are the most prominent. The *DRD2* gene (11q23) is mainly expressed in the striatum and its role is related to several cognitive processes, such as intelligence, learning from errors, and creativity (Reuter *et al.*, 2006; Klein *et al.*, 2007; Shaw, 2007). A functional polymorphism in the promoter region, −141CIns/Del has been associated with alcohol dependence (Lee *et al.*, 2013), schizophrenia (Arinami *et al.*, 1997) and clinical response to antipsychotics (Lencz *et al.*, 2006). The *DRD4* gene (11p15.5) is widely expressed in the central nervous system, mainly in regions related to planning and reward, such as the prefrontal cortex, hippocampus, and amygdala (Meador-Woodruff *et al.*, 1996; De La Garza 2nd and Madras, 2000; Simpson *et al.*, 2010). The main polymorphism studied is a 48 bp VNTR in exon 3, which involves nine different alleles; however, the 4-, 2-, and 7- repeat alleles are the most prevalent (Chang *et al.*, 1996). This polymorphism has been associated with attention deficit hyperactivity disorder (Camarena *et al.*, 2007), obsessive compulsive disorder (Walitza *et al.*, 2008), autism spectrum disorders (Faraone *et al.*, 2005), impulsivity (Eisenberg *et al.*, 2007) and prosocial behaviors (Bakermans-Kranenburg and van Ijzendoorn, 2011).

Musical characteristics require social interaction and cognitive processes, presupposing that the core genes related to different behaviors must influence musical phenotypes. We hypothesized that polymorphisms in the six genes mentioned above, which are associated with different social and cognitive behaviors, could also be related to musical traits. Our aim was to investigate the influence of the *AVPR1A, SLC6A4, ITGB3, COMT, DRD2*, and *DRD4* polymorphisms on musicality in a sample of children from Porto Alegre, Brazil, and thus contribute to understanding the genetic basis of musical aptitude.

## Subjects and Methods

### Sample

Fifty-five students with no musical education from two public schools in Porto Alegre, Brazil, participated in the study. The students were between 7 and 9 years of age and in the 1st to 4th grade of elementary school. This study was approved by the Ethics Committee of the Hospital de Clínicas de Porto Alegre (protocol 10-0562), and all of the children's parents received information about the proposal, since they signed an informed consent form.

Interviews, musical assessments, and collection of biological samples were performed by trained music therapists. Children who had listening difficulties or any restriction to sounds or noises were excluded from the study. All participants exhibited typical development.

### Musical Assessment

Musicality was measured through an individualized music therapy assessment profile (IMTAP) (Baxter *et al.*, 2007), which had its Brazilian version validated (da Silva *et al.*, 2013). The IMTAP assesses functional abilities for each participant through musical activities performed by trained music therapists. Among 10 behavioral and functional domains that could be evaluated independently, the musicality domain, in particular, was assessed in this study. The musicality domain examines the individuals’ innate responses to various musical activities and their ability and desire to participate in each activity. Each IMTAP domain is divided into several sub-domains which help to clearly define the skill sets that are being addressed within the broader domain. The sub-domains of musicality include: fundamentals, tempo, rhythm, dynamics, vocality, perfect and relative pitch, creativity and development of musical ideas, and accompaniment.

IMTAP assessment is done in an objective way. The musicality score of each participant is based in points. The scoring, explained in detail below, is based on the number of times the skills described above are exhibited. All individuals were assessed by the same two music therapists. Although the music therapists were aware that a genetic study would be performed in a posterior step, they had no information on the genetics results. Note that the first objective of IMTAP assessment in our sample was the validation of the instrument in Brazil, excluding any possible biases related to a genetic study.

**Figure 1:**
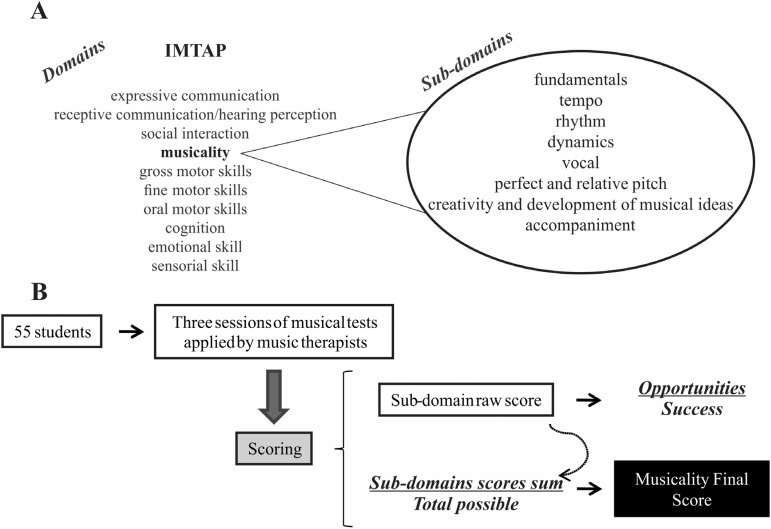
Representation of IMTAP assessment. (A) The IMTAP evaluates 10 musical and/or behavioral domains. In this study, we analyzed the musicality domain, which is assessed through different sub-domains; (B) the scheme for musicality assessment and scoring used in this study.

A structured protocol of musical activities covering all sub-domain abilities was performed for each student. This included three 60 min sessions which were recorded and further analyzed. For the assessment, sessions were scored according to the consistency with which the student presented the skills covered in the musicality sub-domains. The consistency levels were: N = Never, R = Rarely (less than 50%), I = Inconsistent (50–79%) and C = Consistent (80–100%). Counting the number of opportunities given and dividing by the number of times the skill was exhibited, the level of consistency according to this percentage was obtained. Raw scores for sub-domains were calculated based on the number of C, I, R, and N occurrences in all the activities of each sub-domain. The final score for the domain was calculated by dividing the sum of all sub-domain raw scores by the total possible (Figure 1 shows a representation of the IMTAP assessment). The final score for the domain was calculated by averaging the scores given by the two music therapists, and it is also reported as percentage.

### Genotyping

Saliva samples from all 55 students were collected with the Oragene kit (DNA Genotek, Canada), and DNA extraction was performed in accordance with the manufacturer's protocol.

We genotyped both the highly variable microsatellites RS1 and RS3, which are located in the promoter region, and the AVR microsatellite in the intron of the *AVPR1A* gene via polymerase chain reaction (PCR), using labeled primers as described in previous studies (Ukkola *et al.*, 2009). PCR products were analyzed using an ABI 3730xl DNA analyzer (Applied Biosystems, USA), and the product sizes were determined using PeakScanner Software v1.0 (Applied Biosystems).

The 5-HTTLPR/rs25531 polymorphism, which is located in the promoter region of the *SLC6A4* gene, was analyzed based on the tri-allelic format (S, L_A_, and L_G_ alleles). PCR and restriction fragment length polymorphism (RFLP) analysis were done in accordance with the techniques previously described by Kaiser *et al.* (2001) and Stein *et al.* (2006).

The polymorphisms in the genes *ITGB3* and *COMT* (rs15908 and Val158Met, respectively) were genotyped using the Taqman allelic discrimination method (Applied Biosystems), in accordance with the manufacturer's protocol.

The -141CIns/Del polymorphism in the promoter region of the *DRD2* gene was analyzed using the PCR-RFLP method, which was previously described by Arinami *et al.* (1997). The main *DRD4* polymorphism, a 48 bp VNTR in the third exon, was analyzed through PCR, in accordance with Roman *et al.* (1999).

### Statistical Analysis

Allele frequencies were calculated through gene counting. Arlequin 3.5.1.2 and GenePop 4.2 were used to check the Hardy-Weinberg equilibrium. In order to analyze the influence of each gene polymorphism on musicality, we grouped the students according to their genotypes, based on: allele frequencies, differential expression, and/or previous results, and then compared the musicality scores obtained for each group. For the RS1, RS3, and AVR microsatellites of *AVPR1A*, we compared carriers of the 4-, 7-, and 5-alleles, respectively, as well as non-carriers. These alleles were selected due to previous literature reports of associations with musical characteristics (Yirmiya *et al.*, 2006; Ukkola *et al.*, 2009; Ukkola-Vuoti *et al.*, 2011). Individuals homozygous for the 5-HTTLPR/rs25531 L_A_L_A_ genotype present a higher *SLC6A4* mRNA expression comparing to individuals presenting the other genotypes (Hu *et al.*, 2006). Therefore, we compared L_A_L_A_ genotype to the other genotypes (SS, SL_G_, SL_A_, L_G_L_G_, and L_G_L_A_). For the rs15908 variant of the *ITGB3* gene we compared C-carriers and non-carriers. The Met allele of Val158Met of the *COMT* gene has been associated with cognitive processes (Shaw, 2007), therefore we compared Met-carriers and non-carriers. For the −141C Ins/Del of the *DRD2* gene, students were grouped into Del-carriers and non-carriers, because the Del allele exhibits reduced gene expression (Arinami *et al.*, 1997). The 7-repeat allele (7R) of *DRD4* VNTR has been associated with several behavioral traits (Ebstein *et al.*, 1996; Faraone *et al.*, 2001), therefore we compared 7R-carriers and non-carriers among the students.

The association between musicality scores (IMTAP assessment) and the polymorphisms evaluated were analyzed through Generalized Linear Model (GZLM). Normal distribution with identity link function or gamma distribution with log link function were used according to the distribution of musicality scores in each group tested for each polymorphism. All analyses were adjusted for age. Students’ age adjustment was performed due to the correlation found (r = 0.43, p = 0.001) with the musicality scores (Figure S1). Considering multiple testing, a significance threshold corrected by Bonferroni considered 0.05/8 = 0.006. Statistical analysis was done in SPSS 18.0 for Windows.

## Results

The children's age varied between 84 months (7 years) and 118 months (9 years and 10 months), with a mean of 97.45 months (about 8 years and 1 month). The male:female ratio was 28:27. The musicality scores varied between 27.60% and 76.60% (mean of 57.12%, standard error of 1.80%).

Table 1 shows the allele frequencies of the polymorphisms of the *AVPR1A, SLC6A4, ITGB3, COMT, DRD2*, and *DRD4* genes analyzed in this study. All polymorphisms, except for the RS1 and RS3 microsatellites of *AVPR1A*, were in Hardy-Weinberg equilibrium.

**Table 1:** Allele frequency of the polymorphisms analyzed in this study.

Gene/polymorphism	Allele	Count	Frequency
*AVPR1A*/ RS1	1 (302 bp)	1	0.0091
	2 (306 bp)	10	0.0909
	3 (310 bp)	55	0.5000
	4 (314 bp)	31	0.2818
	5 (318 bp)	1	0.0091
	6 (322 bp)	8	0.0727
	7 (326 bp)	1	0.0091
	8 (330 bp)	3	0.0273
*AVPR1A*/ RS3	1 (319 bp)	2	0.0182
	2 (321 bp)	1	0.0091
	3 (323 bp)	6	0.0545
	4 (325 bp)	12	0.1091
	5 (327 bp)	12	0.1091
	6 (329 bp)	22	0.2000
	7 (331 bp)	22	0.2000
	8 (333 bp)	16	0.1455
	9 (335 bp)	3	0.0273
	10 (337 bp)	4	0.0364
	11 (339 bp)	5	0.0455
	12 (341 bp)	4	0.0364
	14 (310 bp)	1	0.0091
*AVPR1A*/ AVR	3 (208 bp)	1	0.0094
	4 (210 bp)	15	0.1415
	5 (212 bp)	43	0.4057
	6 (214 bp)	42	0.3962
	7 (216 bp)	4	0.0377
	8 (218 bp)	1	0.0094
*SLC6A4*/ 5-HTTLPR	S	45	0.4091
	L_A_	52	0.4727
	L_G_	13	0.1182
*ITGB3*/ rs15908	A	66	0.6000
	C	44	0.4000
*COMT*/ Val158Met	Val	63	0.5833
	Met	45	0.4167
*DRD2*/ −141CIns/Del	Ins	89	0.8091
	Del	21	0.1909
*DRD4*/ VNTR Exon 3	2R	8	0.0727
	3R	3	0.0273
	4R	67	0.6091
	5R	1	0.0091
	6R	4	0.0364
	7R	26	0.2364
	8R	1	0.0091

The allele labels in the RS1, RS3, and AVR microsatellites are in accordance to those used in Yirmiya *et al.* (2006).

The results from association analyses between gene polymorphisms and musicality scores are shown in Table 2. We found a nominal association between the RS1 microsatellite of *AVPR1A* and musicality scores. Individuals carrying allele 4 showed higher musicality scores compared to non-carriers (p = 0.038) (Table 2). Other polymorphisms evaluated were not associated with musicality scores (Table 2).

**Table 2:** Results of analyses for association between musicality scores and polymorphisms.

Gene/ polymorphism	Genotype	N	Musicality score^a^	p
*AVPR1A*/ RS1	4/-	24	60.83 (2.38)	0.038
Others^b^	32	54.24 (2.09)		
*AVPR1A*/ RS3	7/-	18	61.31 (3.19)	0.074
Others^c^	37	54.70 (1.97)		
*AVPR1A*/ AVR	5/-	36	56.24 (2.00)	0.734
Others^d^	18	57.45 (2.91)		
*SLC6A4*/ 5-HTTLPR	L_A_L_A_	12	57.49 (3.48)	0.904
Others^e^	43	57.01 (1.84)		
*ITGB3*/ rs15908	AA	18	54.04 (2.86)	0.250
C/-	37	58.24 (2.13)		
*COMT*/ Val158Met	Val/Val	17	60.39 (2.88)	0.193
Met/-	37	55.86 (1.95)		
*DRD2*/ −141CIns/Del	Ins/Ins	36	57.33 (2.13)	0.703
Del/-	19	55.97 (2.86)		
*DRD4*/ VNTR Exon 3	7R/-	18	57.90 (2.57)	0.694
Others^f^	37	56.59 (2.10)		

a Mean (Standard Error);

b All genotypes not containing allele 4;

c All genotypes not containing allele 7;

d All genotypes not containing allele 5;

e Genotypes: SS, SLG, SLA, LGLG, and LGLA;

f All genotypes not containing allele 7R.

## Discussion

Music has played a central role in human history. It is intrinsic to all cultures, both past and present. Although the origins and adaptive functions of music remain unknown, many authors suggest that human abilities to appreciate and practice music may be a biological adaptation, considering that in a long evolutionary past it may have been essential for survival and reproduction (McDermott and Hauser, 2005; Wang, 2015; Honing *et al.*, 2015). Indeed, evidence for signatures of positive selection for abilities that contribute to musical aptitude has been demonstrated, and a possible shared genetic and evolutionary background between music and language is suggested (Liu *et al.*, 2016). A conceivable reason to explain this positive selection could be the reward value of music, in which increased dopamine secretion would be stimulated during musical activities (Salimpoor *et al.*, 2011; Liu *et al.*, 2016).

All humans inherit an intrinsic form of musicality, in which genetic factors may play an important role in its expression. Therefore, genetic studies of musical abilities will enable us to better understand important questions about the origins and selective pressure of music in human history. Although limited, these studies have emerged in recent years, and with increased research efforts, a clearer understanding of the genetic basis underlying musical abilities may emerge (Tan *et al.*, 2014). In this study, we investigated a possible association between musicality and polymorphisms in the *AVPR1A, SLC6A4, ITGB3, COMT, DRD2*, and *DRD4* genes, which are known to influence behavioral and cognitive functions. We found a nominal association between allele 4 of RS1 microsatellite of *AVPR1A* gene and higher musicality scores.

The hormone arginine vasopressin (AVP) and its receptor AVPR1A have a key role in controlling cognitive functions such as memory and learning. The *AVPR1A* gene has been associated with music memory (Granot *et al.*, 2007), music perception (Ukkola *et al.*, 2009), and music listening (Ukkola-Vuoti *et al.*, 2011). Our results add to the evidence of the possible role of *AVPR1A* in influencing music traits. Significant associations between *AVPR1A* microsatellites and musical characteristics have been reported by different authors (Bachner-Melman *et al.*, 2005; Granot *et al.*, 2007; Ukkola *et al.*, 2009; Ukkola-Vuoti *et al.*, 2011). Since *AVPR1A* plays a central role in controlling cognitive functions, modulating social behavior, and influencing memory and learning, these studies strongly suggest a link between different musical activities and human cognitive social skills (Tan *et al.*, 2014). Our results, together with these previous reports, support the idea that *AVPR1A* acting in the neurobiological pathways affecting human social functioning, may modulate important facets of musical traits (Bielsky *et al.*, 2004; Egashira *et al.*, 2007; Ukkola *et al.*, 2009; Tan *et al.*, 2014).

The gene selection for this study was based on previous reports indicating genes associated with musical, behavioral, and/or cognitive traits. The *SLC6A4* gene has been associated with music memory (Granot *et al.*, 2007) and choral singing (Morley *et al.*, 2012). Other genes, such as *COMT, TPH1, DRD2, DRD4, DRD5, IGF2*, and *MAOA* have also been investigated but results have so far been inconclusive. Musical aptitudes comprise a complex network involving a number of genes. These previous studies demonstrating gene associations with musical traits may reflect social communication, emotion, courtship, and other behavioral elements of musical phenotypes (Bachner-Melman *et al.*, 2005; Ukkola *et al.*, 2009). Additionally, AVP and serotonergic and dopaminergic systems share strong functional interactions (Ferris *et al.*, 1997; Seo *et al.*, 2008; Skuse and Gallagher, 2011). Thus, the genes studied here belong to an interactive network involved in several behavioral and cognitive functions. Although some studies have indicated that *SLC6A4* has a role in musical traits, we failed to demonstrate significant association between this gene and musicality scores. Besides, *ITGB3, COMT, DRD2* and *DRD4* roles in musical features were also not demonstrated here. Future investigations, using a greater sample size, may demonstrate new insights in which genes are modulating this phenotype.

Most genetic studies of music traits have investigated each musical ability separately (*e.g.*, music memory, music listening, music perception, singing, dance, or others). Thus, there is a need for a more comprehensive investigation of musical abilities, using an approach that includes a broader range of evaluated skills. Given this scenario, we here analyzed musicality in a more complete way, evaluating musical talent in eight domains of musical traits (fundamentals, tempo, rhythm, dynamics, vocality, perfect and relative pitch, creativity and development of musical ideas, and accompaniment). Nevertheless, musicality is a multifactorial characteristic involving a complex interaction of physical, emotional, cognitive, and psychosocial traits (Levitin, 2012) that challenge its definition and research. Musicality could be investigated in future studies covering other additional features that have not yet been addressed. In addition to the broader characterization of musicality, another singularity in our study was the sample selection. The group studied was quite homogenous, consisting of children that were not attending musical classes, and most of them did not have musical relatives. This differentiates our study from other studies that use either markedly different groups (with and without certain skills) or groups including musical families. Thus, we were able to minimize external influences caused by environmental factors.

The main limitation of our study was the small sample size. However, the musicality assessment was done in a broader manner, using the IMTAP instrument. IMTAP is very extensive. Its use requires a long period of time for musical activities with each participant, for musicality assessment through the analysis of all records, and for subsequent scoring. Therefore, we evaluated a smaller number of children in order to make IMTAP applicable and, consequently, to assess musicality in a unique manner (up until now). Notwithstanding, our sample size may have prevented the detection of small effects, which are related to common genetic variants. Likewise, the statistical power was impaired due to this limitation.

As mentioned, the genotype distribution for the RS1 and RS3 microsatellites of *AVPR1A* deviated from the Hardy-Weinberg equilibrium. This observed deviation is most likely attributed to random chance, due to our small sample size and the presence of several rare alleles in these two microsatellites. Studying these highly polymorphic loci in a small sample makes it easier to observe a departure from expected genotype frequencies, such as we did.

Although many studies have addressed music from a biological perspective, discordant points of view could be considered. Some authors described music merely as technology rather than an adaptation, a pleasure-producing substance useless for biological evolution (Pinker, 1997; reviewed in Huron, 2001 and Honing *et al.*, 2015). Notwithstanding, recent studies have highlighted different genetic variants involved in musical skills, suggesting the important contribution of biology in this behavior. Although the elucidation of a genetic basis underlying musical characteristics is very challenging due to the multifactorial features, a number of studies have yielded important insights. Replication of these results in different studies is required to confirm the findings. In view of the great complexity of factors influencing musical aptitudes, we propose that our suggestive results for the *AVPR1A* gene point to the communicative and social behavioral facets of musical abilities.

In conclusion, our results indicate a contribution from the RS1 polymorphism of the *AVPR1A* gene towards obtaining higher musicality scores. This study is one of the first to investigate musicality in a comprehensive way, evaluating a number of musical aptitudes jointly. We provided an additional investigation on genetic factors influencing musical traits and we expect to contribute to a deeper understanding of the complex role of genes influencing this phenotype.

## Supplementary Material

The following online material is available for this article:

Figure S1 - Correlation between students’ age and musicality scores
