# Supplementary figures and images for: Music genetics research: Association with musicality of a polymorphism in the AVPR1A gene
Source: Genet Mol Biol. 2017 May 22;40(2):421–9. doi: 10.1590/1678-4685-GMB-2016-0021 (PMC5488451; doi:10.1590/1678-4685-GMB-2016-0021)

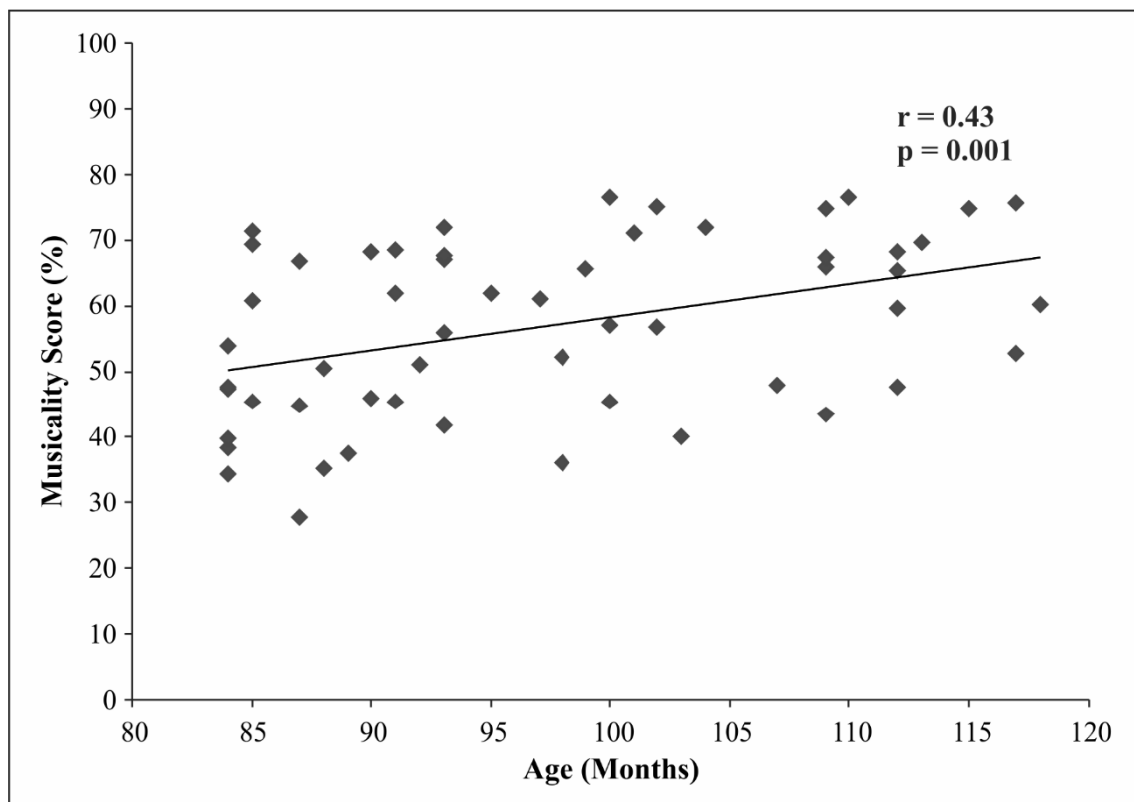

**Figure S1** – Correlation between students' age and musicality scores.

Supplement: Supplementary file 1 [file 1415-4757-gmb-1678-4685-GMB-2016-0021-Suppl01.pdf]
